# Supplementary material for: Force-driven reversible liquid–gas phase transition mediated by elastic nanosponges
Source: Nat Commun. 2019 Jun 17;10:2559. doi: 10.1038/s41467-019-10511-7 (PMC6572794; doi:10.1038/s41467-019-10511-7)
Supplement: Supplementary file 1 — Supplementary Information [file 41467_2019_10511_MOESM1_ESM.pdf]

Supplementary Information

Force-driven reversible liquid–gas phase transition mediated by elastic nanosponges

Nomura *et. al*

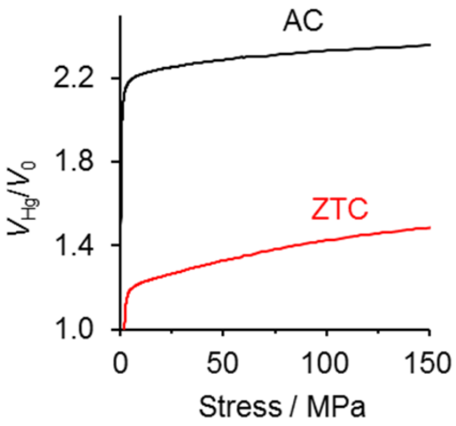

Supplementary Figure 1. Stress-strain curve obtained for ZTC and AC. V_{Hg} and V_0 are the volume change of mercury and initial sample volume, respectively. At low pressure (< 20 MPa), V_{Hg} quickly decreases by Hg impregnation of the interparticle spaces. The bulk modulus (K [MPa]) of the sample can be calculated from the linear part of the stress–strain curve at higher pressure (50–150 MPa), according to equation (2). The bulk moduli of ZTC and AC were determined to 0.70 and 1.7 GPa, respectively (the last value is considered an underestimate because of the presence of larger pores[1]).

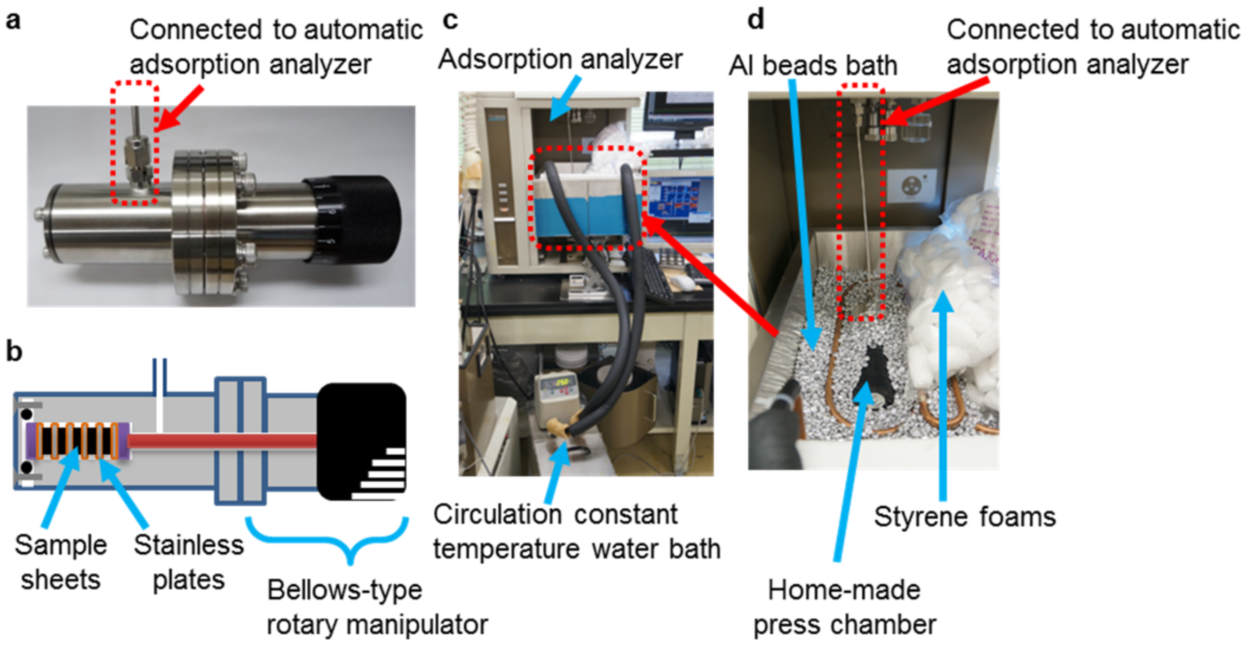

Supplementary Figure 2. Illustrations of the equipment for *in situ* adsorption measurement under pressing. a A photo of a home-made press chamber. **b** An illustration of (**a**) showing the inside. Sample sheets are sandwiched by stainless plates and they are stacked to ensure the sample amount enough for the measurement. The sample stack can be mechanically pressed by a rotary manipulator. The chamber is connected to an automatic adsorption analyzer. **c** A photo of a whole measurement setup. The press chamber is immersed in a constant temperature bath highlighted by a red dashed square. The temperature is maintained by a circulation constant temperature water bath. **d** A photo of the inside of the constant temperature bath. Al beads are used as a heat medium. The press chamber is immersed in the Al beads, together with a coil for circulation temperature control. The Al beads bath is covered by styrene foams. Note the press chamber is intentionally exposed for better view in (**d**).

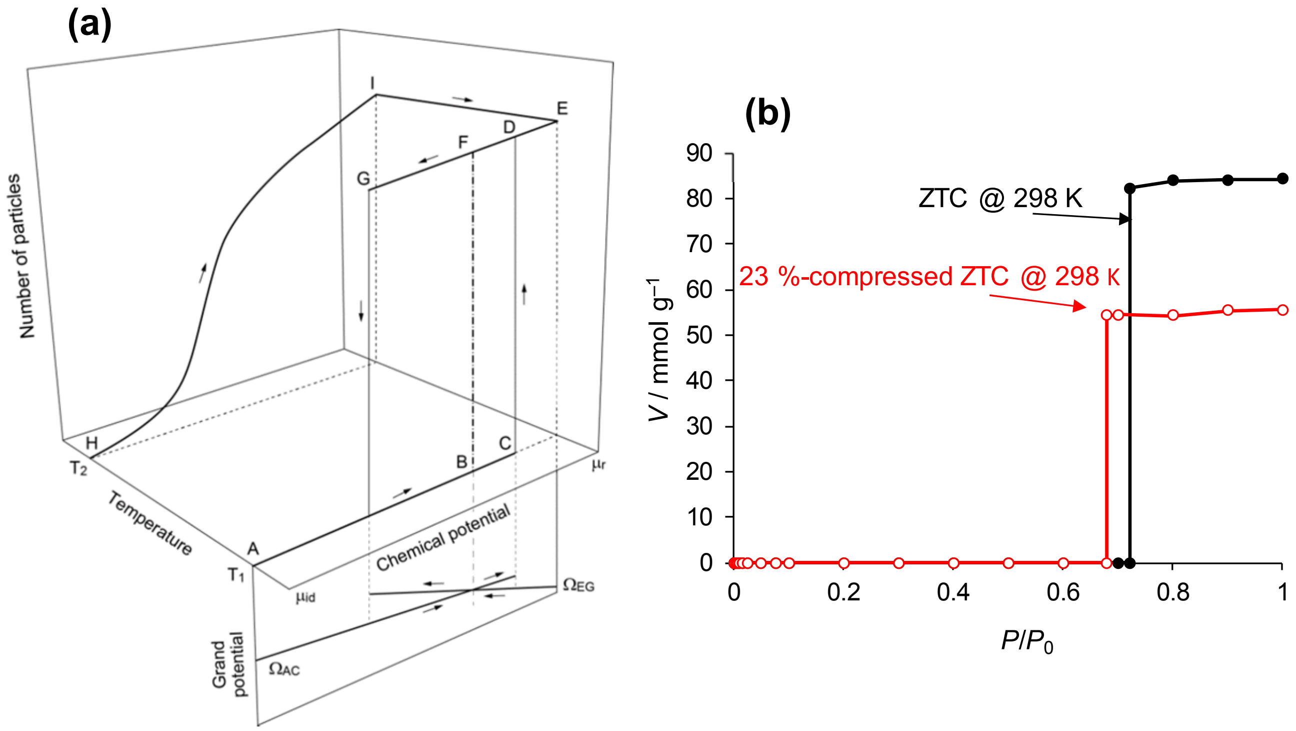

Supplementary Figure 3. Grand canonical Monte Carlo (GCMC) simulation for the H_2O adsorption isotherms (298 K) on ZTC with and without compression. a Schematic representation showing the paths considered in the PG method. The vertical line BF represents the equilibrium vapour-liquid coexistence in a nanopore at subcritical temperature T_1, where the grand potentials, Ω_{AB} and Ω_{DH}, are equal. **b** H_2O adsorption isotherms on the ZTC model containing oxygen-functional groups (Figure 2a) with and without compression.

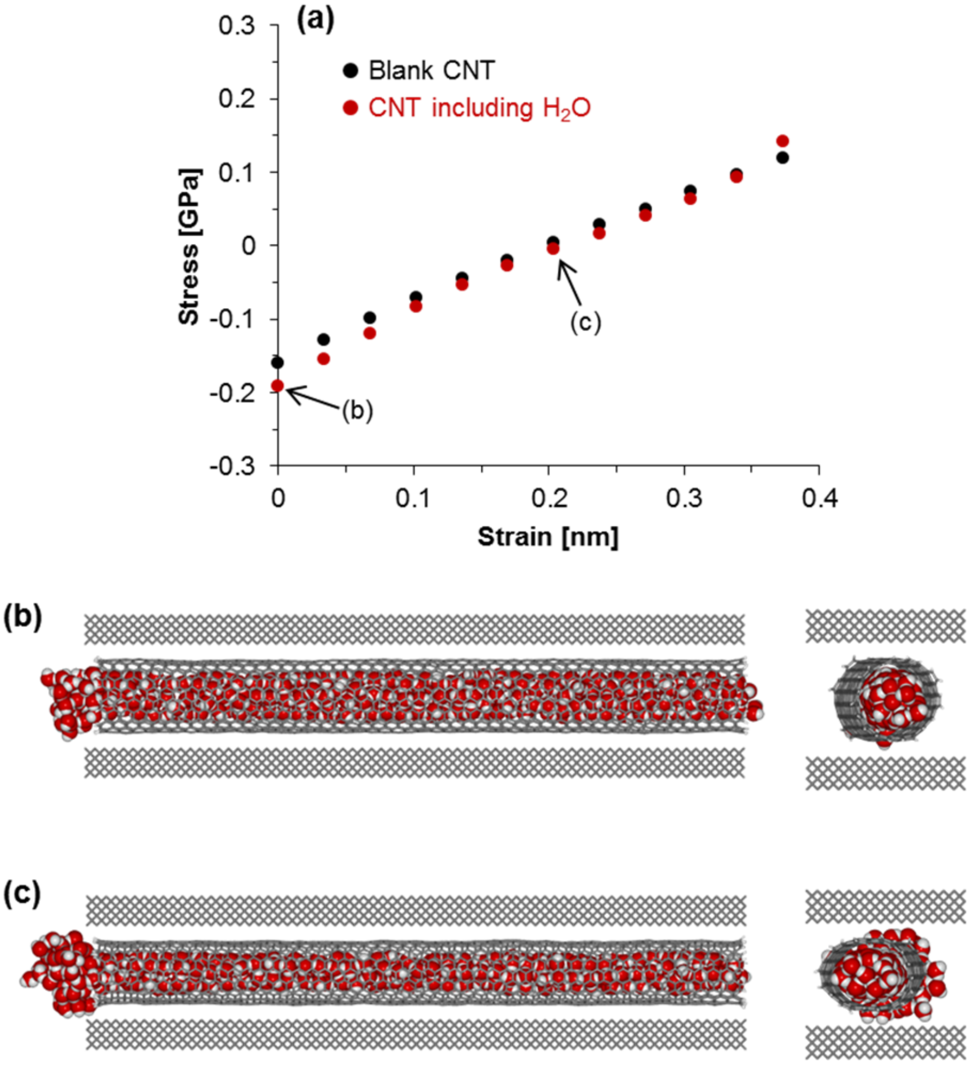

Supplementary Figure 4. Stress-strain curves of a carbon nanotube (CNT) with and without inclusion of H_2O. (a) Stress-strain curves obtained by the MD simulation. A (10,10) CNT is used and its diameter is 1.36 nm which is close to the pore width of ZTC (1.4 nm as center-to-center distance of two pore walls). The Young's modulus of CNT is calculated as 1.2 GPa, close to that of ZTC (0.88 GPa). (b,c) Snapshots of the MD simulation at the points indicated in (a). It is found that the stress-strain curve is not significantly changed by the inclusion of H_2O.

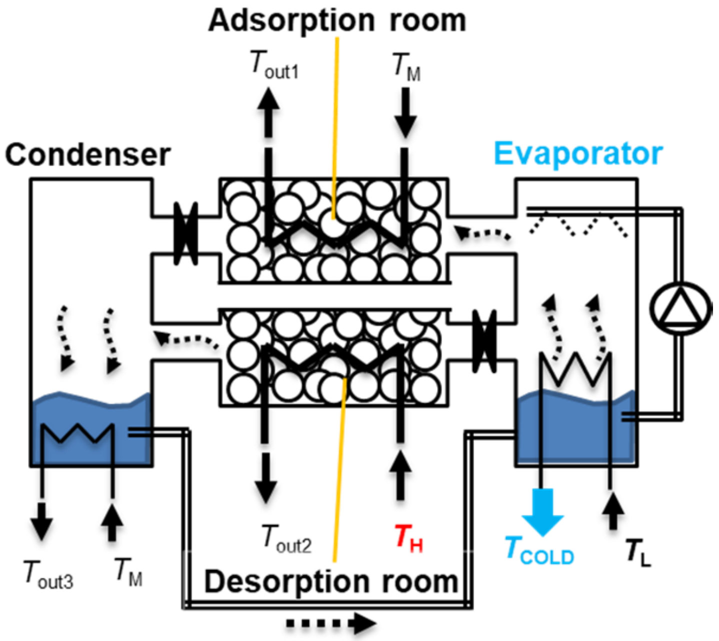

Supplementary Figure 5. A schematic illustration of Adsorption Heat Pump (AHP). A liquid refrigerant is turned into vapour in an evaporator, and by the heat of evaporation, the temperature (T_L) of a target room is cooled down to T_{COLD}. T_L is usually about 286 K for water system. The vapour is then adsorbed in porous material accompanied by the generation of heat of adsorption (exothermic) at an adsorption room. To keep the temperature of the adsorption room constant, an external middle-temperature heat source (T_M) is used. T_M is usually about 303 K for water system. When the adsorbed amount reaches a certain level, the adsorption room is switched to be a desorption room. Then, the refrigerant is desorbed (endothermic) by using an external high-temperature heat source (T_H). T_H is usually about 350 K for water system. The vapour is then translated to liquid at a condenser, accompanied by the heat of condensation (exothermic). To keep the temperature of the condenser constant, an external middle-temperature heat source (usually the same as T_M) is used. Then, the liquid is transferred to the evaporator. In AHP, cooling is thus done by the evaporator, and therefore, AHP is categorized to the Refrigeration based on the Bulk Phase Transition (RBPT). Loosely speaking, adsorption and desorption in AHP play a role of mass transfer instead of a compressor and an expansion valve in air conditioners. AHP enables the use of water as a refrigerant, whereas AHP is much bulky than conventional air conditioners because of the limited adsorption capacity of nanoporous solids operated around P/P_0 = 0.1–0.5, and has not been used for compact air conditioners for private rooms and automobiles.

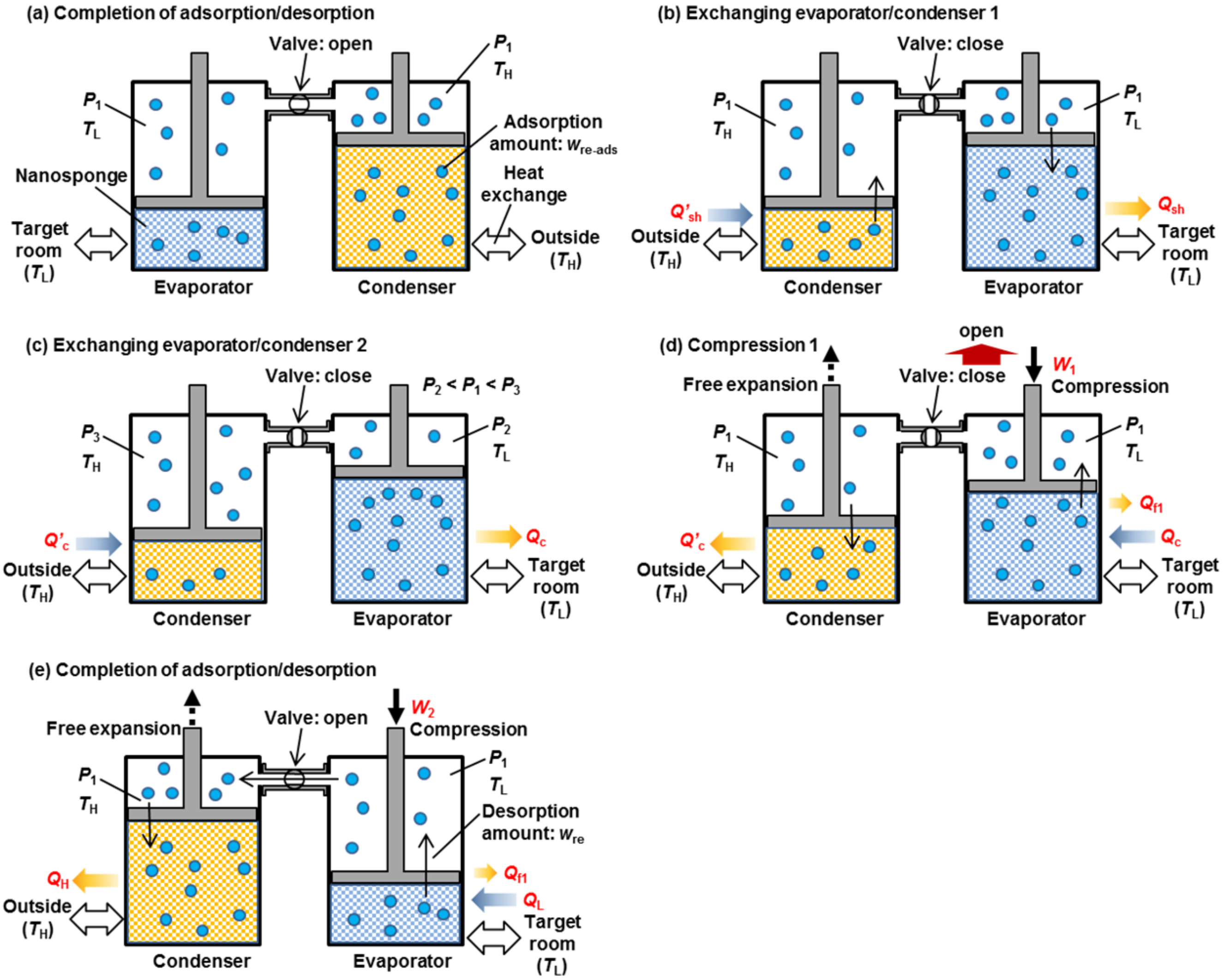

Supplementary Figure 6. Continuous operation of the prototype system of Refrigeration based on the Mechanical-force-induced Phase-Transition of Adsorbate (RMPTA) shown in Fig. 1e-i. a The state at the completion of adsorption/desorption. The nanosponge in the evaporator is compressed up to the limit deformation, and the nanosponge in the condenser is not compressed and stays at its original porosity. **b** The moment upon exchanging the evaporator and the condenser each other. First, a valve between the two chambers is closed, and then the heat reservoirs (a target room and an outside in **a**) of the two chambers are replaced each other. The temperature of the evaporator is changed from T_H to T_L, while the temperature change of the condenser is opposite. Upon changing the temperature, adsorption/desorption occurs at the evaporator/condenser respectively. For simplifying, it is assumed here that adsorption/desorption starts upon the completion of the temperature change. By the temperature change, sensible heats, Q_{sh} and Q'_{sh}, are transferred from the evaporator to the target room and from the outside to the condenser, respectively. **c** The moment upon the completion of adsorption/desorption from the state of **b**. During the adsorption/desorption, subliminal heats, Q_c and Q'_c are transferred from the evaporator

to the target room and from the outside to the condenser, respectively. Upon the completion of adsorption/desorption, compression of the nanosponge in the evaporator is started, while free-expansion of the nanosponge in the condenser is started. **d** The moment at which the vapour pressures of the two chambers become P_1. At this moment, the valve is opened. The work applied to the evaporator from **b** to **c** is W_1 [J], while subliminal heats, Q_c and Q'_c are transferred from the target room to the evaporator and from the condenser to the outside, respectively. When the nanosponge is not perfectly elastic, a part of W_1 turns into heat (Q_{f1}) by the internal friction. Also after the valve is open, the compression of the nanosponge successively continues. **e** The state at the completion of adsorption/desorption. The work loaded from **d** to **e** is W_2 [J], while a possible heat loss by the internal friction is Q_{f2}. The subliminal heats generated at the evaporator and the condenser during this period are Q_L and Q_H, respectively. The total work (W_{ns}) applied to the nanosponge is obtained by $W_{ns} = W_1 + W_2$, while the total heat loos by the internal friction (Q_f) is obtained by $Q_f = Q_{f1} + Q_{f2}$. See Supplementary Methods for the calculation of COP in this system.

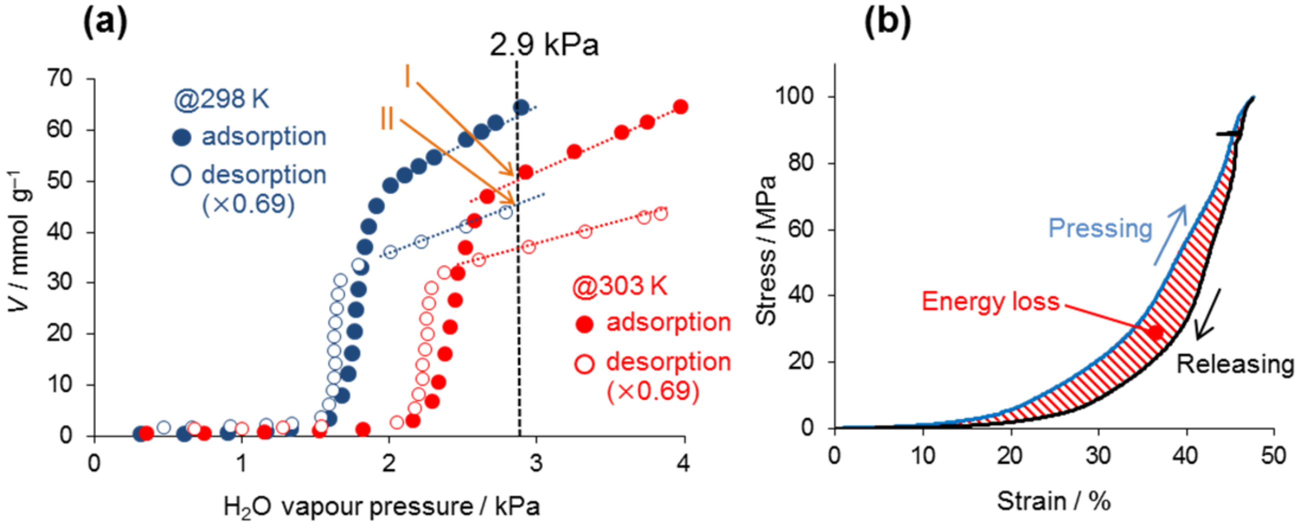

Supplementary Figure 7. Water vapour adsorption/desorption isotherms and stress-strain curves of ZTC/PTFE sheets for the estimation of COP. a The H_2O vapour adsorption isotherms were measured on the pristine ZTC at 298 (T_L) and 303 K (T_H), respectively. The isotherms on the compressed ZTC were calculated by multiplying 0.69 to the data on the pristine ZTC, based on the result in which the adsorption amount becomes 0.69 of its original value by pressing with 83 MPa (Fig. 4d). From the deformation and the pressure, W_{ns} in equation (23) is calculated as 18.5 kJ kg^{-1}. **b** Stress-strain curves measured on a ZTC/PTEF sheet. By using these data, COP is calculated at the vapour pressure of 2.9 kPa, based on equation (23). In this equation, $\Delta_{vap}H$ and c_{re} are 2453 kJ kg^{-1} and 4.184 kJ kg^{-1} K^{-1}, respectively. $T_H - T_L$ is 5 K. c_{ns} can be approximated by the value of the graphite (0.72 kJ kg^{-1} K^{-1}). From equation (25), $V_0 = w_{ns}/\rho_{ns\text{-}ap} = 0.001976w_{ns}$. $w_{re\text{-}ads}$ can be expressed by w_{ns}, from the adsorption amount at 303 K on the pristine ZTC (point I; 51.1 mmol g^{-1} = 0.92 kg kg^{-1}) as $0.92w_{ns}$. As described in equation (28), w_{re} is the difference of $w_{re\text{-}ads}$ and w_e. w_e can be expressed by w_{ns}, from the adsorption amount at 298 K on the compressed ZTC (point II; 42.8 mmol g^{-1} = 0.77 kg kg^{-1}) as $0.77w_{ns}$. Therefore, $w_{re} = 0.92w_{ns} - 0.77w_{ns} = 0.15w_{ns}$. From the area highlighted in (**b**), the energy loss ratio (φ_f) by the internal friction of ZTC is calculated as 0.25. The energy loss turns into heat and warms up the target room to be cooled. Accordingly, COP for ZTC working between 298 and 303 K at 2.9 kPa can be calculated as 17.3.

Properties of the imaginary nanosponge

Pore volume (V_{ms}) = 1.17×10^{-3} m^3 kg^{-1}
The ratio of the pore volume filled with refrigerant (φ_{ns}) = 1
True density (ρ_{ns}) = 2.00×10^{-3} m^3 kg^{-1}
Pore diameter: 1.2 nm
Young's modulus (E) = 0.27 GPa
Young's modulus (E) = 0.38 GPa
Specific heat capacity (c_{ns}) = 0.72 kJ kg^{-1} K^{-1}
Energy loss ratio (φ_f) = 0.25

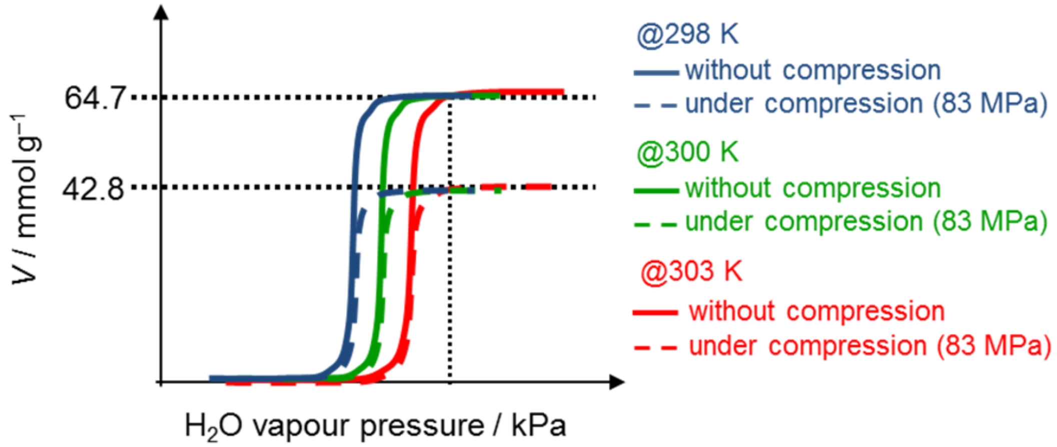

Supplementary Figure 8. Example of adsorption isotherms on an imaginary nanosponge. The properties of the nanosponge are given as an inset. V_{ns} and E are the same as those of ZTC, while ρ_{ns} and c_{ns} are the same as those of graphite. Thus, the imaginary nanosponge is not far from actual materials. Based on equation (23), larger w_{re} affords better COP. Therefore, the gap in the adsorption values at the points I and II shown in the Supplementary Fig. 7a should be larger. Herein, an imaginary adsorption isotherms are shown, which is obtained by modifying Supplementary Fig. 7a, by the following two assumptions: (i) at 303 K and 2.9 kPa, the adsorption amounts in the pristine and compressed ZTCs are the same as those at 298 K, and (ii) the isotherms have a flat plateau after water uptake is completed. The latter feature is seen in mesoporous silicas with hydrophilic uniform mesopores[2], and can be considered as an achievable target also in elastic nanosponge materials. Additionally, the H_2O uptake pressures at different temperatures are adjusted according to the fact that the H_2O uptake occurs at the same P/P_0 regardless of temperature[2]. Consequently, the isotherms shown here can be an achievable target. In this case, w_{re} is 0.36w_{ns}, and $w_{re\text{-}ads}$ = 1.17w_{ns}. The work W_{ns} in equation (23) can be experimentally obtained as explained in Supplementary Figure 7. Therefore, COP is estimated as 43.3 regardless of ΔT, further highlighting the great potential of RBPT.

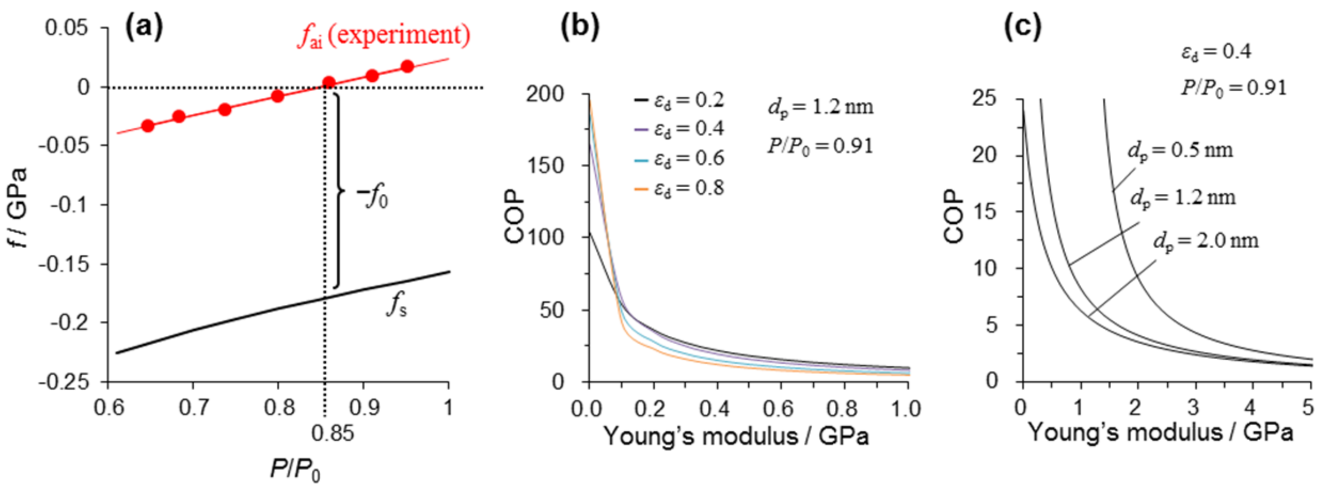

Supplementary Figure 9. Estimation of the relation between COP and *E*. a The estimation of f_0 by comparing f_{ai} obtained by experiment and f_s which can be calculated by equation (21). f_0 can be determined as the difference between f_{ai} and f_s when the deformation of nanosponge becomes 0. In the case of ZTC, f_0 is −0.18 GPa at P/P_0=0.85. **b** The relation between COP and the Young's modulus (*E*) of nanosponge at the very small *E* region. **c** The relation between COP and *E* for several different pore sizes (d_p) within 2 nm, in which water-vapour adsorption occurs in porous carbons. ε_d and P/P_0 are fixed to be 0.4 and 0.91, respectively.

Supplementary Table 1. Bulk moduli (*K*) of conventional materials.

Category	Material	Pore size / nm	K^a / GPa	E^b/ GPa	Reference
Metal-organic	ZIF-8	~1.1	9.2	3.0	Ito, M. *et al. Chem-Eur J* **19**, 13009-13016, (2013). Tan, J. C. *et al.* PNAS, 107, 9938-9943 (2010).
frameworks	MOF-C30	~2	4.11	–	Han, S. S. & Goddard, W. A. *J Phys Chem C* **111**, 15185-15191, (2007).
	MOF-5	~1.5	17	2.7	Tan, J. C. & Cheetham, A. K. *Chem Soc Rev* **40**, 1059-1080, (2011). Bahr, D. F. & Reid, J. A. *Physical Review B* **76**, 184106-184112 (2007).
Porous silicas	SBA-15	~9	12.0	–	Kizzire, D. G. *et al. Micropor Mesopor Mat* **252**, 69-78, (2017).
	FDU-12	~20	5.5	–	Mayanovic, R. A. *et al. Micropor Mesopor Mat* **195**, 161-166, (2014).
Zeolites	Zeolite Y (FAU)	~1.2	13	37.5	Ito, M. *et al. Chem-Eur J* **19**, 13009-13016, (2013). Charitidis, C. A. *et al.*, *Thin Solid Films,* **526**, 168-175 (2012).
	Natrolite (NAT)	0.45	48.5	77.9	Sanchez-Valle, C. *et al.*, *J. Appl. Phys*. **98**, 053508 (2005).
	Phillipsite (PHI)	~0.6	67	–	Gatta, G. D. & Lee, Y. *Micropor Mesopor Mat* **105**, 239-250, (2007).
Covalent organic	COF-102	~2.7	21.6	~22.9	Zhou, W., Wu, H. & Yildirim, T. *Chem Phys Lett* **499**, 103-107, (2010).
frameworks	COF-108	~2.8	4.9	~5.5	
Metals	Al	nonporous	75.5	70	James, A. M. & Lord, M. P. (Macmillan Press, 1992).
	Ag	nonporous	103.6	83	
	Au	nonporous	217	78	
Polymers	Polystyrene	nonporous	3.9-6.0	3-3.5	Bondi, A. A. (Wiley, 1968).
	Polymethyl methacrylate	nonporous	3.7-6.3	2.4-3.4	
	Polyvinyl chloride	nonporous	4.2-5.6	2.4-4.1	

[a] Bulk modulus. [b] Young's modulus.

Supplementary Table 2. Parameters for the TIP4P water model.

Parameters	Values
q_{H} (e)	+0.520
q_{O} (e)	0.0
q_{M} (e)	−1.04
ε_{O} (kJ/mol)	0.64895
σ_{O} (Å)	3.154
r_{OH} (nm)	0.09572
r_{OM} (nm)	0.015
Θ_{HOH} (deg)	104.52

Supplementary Table 3. LJ parameters for the ZTC models.

Element	σ (nm)	ε (kJ/mol)
C	0.3431	0.440
H	0.2571	0.184
O	0.3118	0.251

Supplementary Table 4. LJ parameters for the (10,10) nanotube and the diamond slab.

atom	σ (nm)	ε (kJ/mol)
C	0.340	0.274
H	0.265	0.145

Supplementary Table 5. Parameters for the TIP4P water model.

q_H (e)	+0.520
q_O (e)	0.0
q_M (e)	−1.04
ε_O (kJ/mol)	0.775
σ_O (nm)	0.3154
r_{OH} (nm)	0.09572
r_{OM} (nm)	0.015
Θ_{HOH} (deg)	104.52

Supplementary Table 6. Parameters for conventional RBPT using HFC-134a.[a]

State[b]	Phase	P / MPa	H / kJ kg^{-1}	S / kJ kg^{-1} K^{-1}	T / K	V_m / m^3 kg^{-1}	COP
1	Saturated gas	0.34	250	0.92	277	0.069	
2	Superheated gas	1.0–1.2	272–276	0.92	316–323	0.023–0.027	5.3–
3	Saturated liquid	1.0–1.2	105–114	268–271	312–318	~0.0008	6.6
4	Gas+liquid	0.34	105–114	-	277	-	

[a] See Supplementary Methods for the detailed calculation.

[b] Four states correspond to those in Fig. 6a.

Supplementary Table 7. Parameters for imaginary RBPT using water.[a]

State[b]	Phase	P / kPa	H / kJ kg^{-1}	S / kJ kg^{-1} K^{-1}	T / K	V_m / m^3 kg^{-1}	COP
1	Saturated gas	0.87	2511	9.0	278	147	4.7–5.4
2	Superheated gas	10–13	2940–3004	9.0	504–537	19–23	
3	Saturated liquid	10–13	192–214	0.65–0.72	319–324	~0.001	
4	Gas+liquid	0.87	192–214	-	278	-	

[a] See Supplementary Methods for the detailed calculation.

[b] Four states correspond to those in Fig. 6a.

Supplementary Methods

Grand canonical Monte Carlo (GCMC) simulations for the H_2O-vapour adsorption on ZTC with and without compression

GCMC simulations were performed to calculate H_2O adsorption isotherms on the oxygen-containing ZTC model with and without compression, four trial moves (displacement, rotation, creation, and deletion) for H_2O were executed with the same probabilities, with all framework atoms immobilized. We used a 4.814 × 3.707 × 4.814 nm simulation box for ZTC with compression and a 4.814 × 4.814 × 4.814 nm simulation box for ZTC without compression, with periodic boundary conditions in three directions. The system was equilibrated for 2.5×10^7 Monte Carlo steps, and then data were collected over another 2.5×10^7 steps, which corresponded to more than 1×10^4 trials per H_2O molecule. The sum of H_2O–H_2O and H_2O–ZTC interaction potentials, U, was assumed to be the sum of the Coulombic and Lennard-Jones (LJ) potentials:

$$U = U_{\mathrm{Coulombic}} + U_{\mathrm{LJ}} \tag{3}$$

$$U_{\mathrm{Coulombic}} = \sum \frac{q_{\mathrm{i}} q_{\mathrm{j}}}{4\pi\varepsilon_0 r_{\mathrm{ij}}} \tag{4}$$

$$U_{\mathrm{LJ}} = \sum 4\,\varepsilon_{\mathrm{ij}} \left[\left(\frac{\sigma_{\mathrm{ij}}}{r_{\mathrm{ij}}} \right)^{12} - \left(\frac{\sigma_{\mathrm{ij}}}{r_{\mathrm{ij}}} \right)^{6} \right] \tag{5}$$

where q_{i} is the atomic charge, ε_0 the vacuum permittivity (8.8542×10^{-12} C^2 N^{-1} m^{-2}), r_{ij} the interatomic distance, and σ_{ij} and $\varepsilon_{\mathrm{ij}}$ the LJ parameters. The interaction potentials were calculated with the cross-interaction parameters obtained from the Lorentz-Berthelot mixing rules, and were truncated at a cut-off distance of 1.89 nm. The atomic charges in each of the ZTC models were obtained by the dispersion corrected density functional theory (DFT-D3) calculations with the PBE functional and DZVP-MOLOPT basis set and Mulliken population analyses, using the CP2K software package.[3] We used the TIP4P water model[4] and the universal force field (UFF)[5] was applied to calculate the LJ interaction terms for the ZTC atoms (see Supplementary Table 2 and 3).

At subcritical temperatures, the adsorption isotherm of H_2O on a porous material obtained by the GCMC method exhibits a hysteresis loop formed by discontinuous condensation and evaporation branches, and is not capable of determining the equilibrium phase transition between the vapour-like and liquid-like states. We therefore used the Peterson-Gubbins method[31] to determine the equilibrium phase transition pressure. Typical adsorption isotherm of H_2O on a nanopore by the GCMC method is shown in Supplementary Fig. 3a (lines A-B-C and E-D-F-G). The temperature of the system is T_1, which is below the capillary critical point. The adsorption branch is discontinuous because of a spontaneous condensation from a vapour-like state (point C) to a liquid-like state

(point D). Likewise, the desorption branch contains a spontaneous evaporation from point E to point G. The lines BC and FG correspond to metastable states, and the limiting metastabilities are dominated by energetic barriers separating the vapour-like and liquid-like states that the system should overcome. The vertical line BF represents the vapour-liquid coexistence (equilibrium chemical potential μ_{eq}), and the grand thermodynamic potentials at the points B and F are equal. According to Peterson and Gubbins,[6] the grand thermodynamic potential, Ω_{AC}, along the continuous adsorption branch (line AC) is calculated by integrating the Gibbs adsorption isotherm, N_{AC}:

$$\Omega_{AC}(\mu, T_1) = \Omega_A(\mu_{id}, T_1) - \int_{\mu_{id}}^{\mu} N_{AC}(\mu', T_1) d\mu' \quad (6)$$

where μ is a given chemical potential. The first term in the right-hand side is the grand thermodynamic potential at chemical potential μ_{id}, which is low enough so that the adsorption amount N_A at point A is essentially the ideal gas value:

$$\Omega_A(\mu_{id}, T_1) = -kT_1 N_A(\mu_{id}, T_1) \quad (7)$$

where k is the Boltzmann constant. The grand thermodynamic potential Ω_{EG} along the desorption branch (line EG) is calculated by integrating the Gibbs adsorption isotherm N_{EG} from point E at chemical potential μ_r. Point E can be arbitrarily chosen if $\mu_{eq} < \mu_r$. The Ω_E value is obtained by integration along two reversible paths, namely, a supercritical adsorption isotherm N_{HI} at temperature T_2 (line HI), and a path at the constant chemical potential μ_r (line IE), which connects the two isotherms at T_1 and T_2. The integration of the supercritical adsorption isotherm from μ_{id} (point H) to μ_r (point I) is expressed as:

$$\Omega_I(\mu_r, T_2) = -k_2 T_2 N_H(\mu_{id}, T_2) - \int_{\mu_{id}}^{\mu_r} N_{HI}(\mu, T_2) d\mu \quad (8)$$

and then, the integration along the path IE at the constant chemical potential μ_r from T_2 to T_1 is expressed as:

$$\Omega_E(\mu_r, T_1) = \Omega_I(\mu_r, T_2)\left(\frac{T_1}{T_2}\right) + T_1 \int_{\frac{1}{T_2}}^{\frac{1}{T_1}} [E_{IE}(\mu_r, T) - N_{IE}(\mu_r, T)\mu_r]\, d(1/T) \quad (9)$$

where N_{IE} is the number of particles and E_{IE} is the sum of the potential energy and the kinetic energy ($3NkT/2$) along the line IE. Therefore, the grand thermodynamic potential Ω_{EG} is obtained as:

$$\Omega_{EG}(\mu, T_1) = \Omega_E(\mu_r, T_1) - \int_{\mu_r}^{\mu} N_{EG}(\mu', T_1) d\mu' \quad (10)$$

Finally, the true phase equilibrium is determined as the point of intersection between the grand

thermodynamic potentials, Ω_{AC} and Ω_{EG}.

By the GCMC method, we generated an adsorption isotherm of H_2O at 298 K and a supercritical adsorption isotherm at 700 K, and a path at the constant chemical potential $\mu_r^* = \mu_r/\varepsilon_O = -69.15$, which connects the two isotherms. We performed the GCMC simulations at seven different temperatures to obtain the path at the constant chemical potential of $\mu_r^* = -69.15$: 650 K, 600 K, 550 K, 500 K, 450 K, 400 K and 350 K. The H_2O adsorption isotherms of the ZTC models with and without compression by the GCMC method were obtained as well (Supplementary Fig. 3b).

MD Simulation for stress-strain curves of CNT with and without inclusion of H_2O

Stress-strain curves of nanosponge with and without inclusion of H_2O were calculated by the MD simulation (LAMMPS software package)[7]. A simple (10,10) single-walled carbon nanotube (CNT) was used as a model substrate. CNT (both ends open, terminated by H) was sandwiched by two indenters (diamond slabs). After setting 350 H_2O molecules inside CNT, the system was equilibrated for 100 ps at 298.15 K with fixing the positions of the two slabs. The initial distance between the slabs is L_z = 1.969 nm. The size of simulation box was 16 × 6.6 × 7.0 nm and the periodic boundary conditions were applied in three directions. The time step for integrating equations of motion was set to be 0.5 fs. The AIREBO potential[8] was used as the interatomic force field for the C and H atoms (Supplementary Table 4). The LJ potential was applied for the interaction between CNT and the two diamond slabs. The TIP4P model (see Supplementary Table 5) was assumed for the H_2O molecule and the SHAKE method was used to constrain the bonds of H_2O. The Ewald method was applied to calculate the electrostatic interactions between the H_2O molecules. The cross-interaction parameters for the LJ potentials among H_2O, CNT, and the two slabs were obtained from the Lorentz-Berthelot mixing rules, and truncated at a cut-off distance of 1.3 nm.

After the equilibration stage, the MD simulation in the microcanonical (NVE) ensemble was performed to compress CNT by moving one side of slab to the other with the rate of 6.78×10^{-4} nm ps^{-1}. The instantaneous force, F_z, along the z axis (perpendicular to the slabs) was calculated every 0.5 ps. The obtained forces were divided by the surface area of the slab, A_s, to convert to stress, $\sigma_z = F_z/A_s$, and plotted as a function of the strain of the system. The MD simulation for the compression of the empty CNT was also performed.

To evaluate the stress exerted on the diamond slab in equilibrium state, we performed the NVT-MD simulations using a series of configurations obtained from the above-mentioned NVE-MD simulation. The systems at each L_z were equilibrated for 300 ps by NVT-MD simulation at 298.15 K with fixing the two slabs, and subsequently the ensemble averages of F_z were obtained

during the additional NVT-MD simulations at 298.15 K for 100 ps. The stress-strain curves thus obtained are shown in Supplementary Figure 4a.

MD Simulation for the translation of adsorbed H_2O into H_2O vapour by mechanical pressing

The MD simulation for forced H_2O desorption from ZTC by compression was performed using the LAMMPS software package[7]. We cut out a structure from the 2×2×2 supercell of ZTC model previously developed by Nishihara *et al.*[9] (4.814 × 4.814 × 4.814 nm) and terminated the cutting portions by H atoms. The obtained finite ZTC model was set between two rigid plates (indenters) composed of C atoms together with 3243 molecules of H_2O. The system was equilibrated by the MD simulation in the canonical ensemble at 298 K for 200 ps in such a state that the positions of the two plates are fixed. The size of simulation box was 15 × 15 × 15 nm and the periodic boundary conditions were applied in three directions. The time step for integrating equations of motion was set to be 0.5 fs. The AIREBO potential[33] was used as the interatomic force field for C and H atoms in the finite ZTC model. The LJ potential was applied for the interaction between the finite ZTC model and the two rigid plates, and the interaction parameters were taken from UFF. The TIP4P model was assumed for the H_2O molecules and the SHAKE method was used to constrain the bonds of H_2O. The Ewald method was applied to calculate the electrostatic interactions between the H_2O molecules. The cross-interaction parameters for the LJ potentials among H_2O, the finite ZTC model, and the two rigid plates were obtained from the Lorentz-Berthelot mixing rules, and were truncated at a cut-off distance of 1.3 nm. We finally performed the MD simulation in the microcanonical ensemble for forced H_2O desorption by moving the one rigid plate toward another rigid plate with the rate of 0.00125 nm ps^{-1}. The MD simulation was run for 1 ns and thus the finite ZTC model was deformed by 1.25 nm (compressed to 76%).

To accelerate the forced H_2O desorption, we also performed the MD simulation in the canonical ensemble for 5 ps at 350 K by using the initial finite ZTC model with H_2O as the initial structure, and then switched to the microcanonical ensemble and compressed the finite ZTC model with the rate of 0.002 nm ps^{-1} for 1 ns (compressed to 62%).

Calculation of COP of RBPT using HFC-134a or water

The change from the state **1** to **2** in Fig. 6a is an isentropic process ($S_1 = S_2$). A refrigerant is saturated vapour at the state **1**. It is turned into superheated vapour (state **2**) by a compressor with an external work (W_{in}), which corresponds to the enthalpy change ($H_2 - H_1$). The change from the state **2** to **3** is an isobaric process ($P_2 = P_3$). The refrigerant is turned into saturated liquid by a condenser

at which the refrigerant exhausts the heat (Q_H) to outside of a target room to be cooled. Q_H corresponds to the enthalpy change ($H_3 - H_2$). The change from the state **3** to **4** is an isenthalpic process ($H_3 = H_4$). An expansion valve (Fig. 6a) decreases the pressure of the refrigerant, and turns it into a mixture of gas and liquid. In this process, the evaporation heat and Joule–Thomson effect decrease the temperature. This is an irreversible process. The change from the state **4** to **1** is an isobaric process ($P_4 = P_1$). The refrigerant is turned into saturated vapour by an evaporator at which the refrigerant gains the heat (Q_L) from the target room, meaning that the room is cooled. Q_L corresponds to the enthalpy change ($H_1 - H_4$).

The coefficient of performance (COP) is defined as follows;

$$\mathrm{COP} = \frac{Q_\mathrm{L}}{W_\mathrm{in}} = \frac{H_1 - H_4}{H_2 - H_1} \tag{11}$$

When the pressure (P_1) of a refrigerant at the state **1** is assumed, the corresponding enthalpy of the saturation vapour (H_1) can be determined from the steam table of the refrigerant[10,11]. Then, by assuming the pressure (P_2) at the state **2**, the corresponding enthalpy of the superheated vapour (H_2) is determined as a result of an isentropic change from state **1**. The enthalpy (H_3) of saturated liquid at the state **3** is also determined from the assumption of pressure ($P_3 = P_2$). The enthalpy (H_4) at state **4** is equal to H_3.

In the case of HFC-134a, when the operation pressures at the states **1&4** and **2&3** are 0.34 and 1 MPa, respectively, the temperatures at the states **1&4**, **2**, and **3** are 277, 316 and 312 K, respectively, which are suitable for air conditioners. Thus, Q_L (= $H_1 - H_4$ = 250 – 105 kJ kg^{-1}) can be calculated to be 145 kJ kg^{-1} and the W_{in} (= $H_2 - H_1$ = 272 – 250 kJ kg^{-1}) supplied to the compressor is obtained as 22 kJ kg^{-1}, yielding COP of 6.6. When the pressure at the states **2&3** is increased to 1.2 MPa, Q_L decreases to 136 kJ kg^{-1} and W_{in} becomes 26 kJ kg^{-1}, yielding the COP of 5.3.

In the case of water, when the operation pressures at states **1&4** and **2&3** are 0.87 and 10 kPa, respectively, appropriate temperature range can be achieved for the states **1&4** (278 K) and 3 (319 K). However, the temperature of superheated vapour at the state **2** increases to as high as 504 K. On the other hand, Q_L (= $H_1 - H_4$ = 2511 – 192 kJ kg^{-1}) is calculated as 2319 kJ kg^{-1}, 16 times larger than that of the HFC-134a case. This is because of the much higher latent heat of water (2337 kJ mol^{-1}) than that of HFC-134a (143 kJ mol^{-1}). Despite the great merit in Q_L, W_{in} (= $H_2 - H_1$ = 2940 – 2511 kJ kg^{-1}) supplied to the compressor becomes 429 kJ kg^{-1}, 20 times larger than that of the HFC-134a case. This is because of the very low pressures (0.87–10 kPa), resulting in a very large specific volume (147 m^3 kg^{-1}), about 2100 times larger than that of HFC-134a (0.069 m^3 kg^{-1}). Thus, COP eventually falls in 5.4, almost the same level as that in HFC-134a, along with the

penalty of its huge system volume. This is why water is not used in conventional air conditioners. When the pressures at the states **2&3** are increased to 1.3 kPa, COP decreases to 4.7.

Calculation of COP of RMPTA

Based on the prototype RMPTA system shown in Fig. 6b, a continuous refrigeration cycle can be designed as shown in Fig. 6e and f, and more details are illustrated in Supplementary Fig. 6. Supplementary Fig. 6a is at the state of the completion of adsorption/desorption in condenser/evaporator, respectively. The evaporator and the condenser are thermally connected to a target room (temperature: T_L [K]) to be cooled and an outside (temperature: T_H [K]), respectively, through heat exchangers. For simplifying, the temperatures of the evaporator and the condenser are assumed to be the same as T_L and T_H, respectively. The vapour pressure of the two chambers is P_1 [Pa]. In the next step (Supplementary Fig. 6b), a valve between the two chambers is closed, and the heat reservoirs of the two chambers are exchanged each other. Thus, the temperatures of the condenser and the evaporator become T_H and T_L, respectively. At this point, it is necessary to consider the sensible heat (Q_{sh} [J]) of the refrigerant-including nanosponge (Supplementary Fig. 6b), generated due to the temperature change from T_H to T_L. Q_{sh} is discharged to the target room, and it causes the loss of COP. The contribution of the sensible heat of the vapour phase is much smaller than Q_{sh} and can be ignored. Q_{sh} can be expressed by the following equation using the specific heat capacities of refrigerant (c_{re} [J kg^{-1} K^{-1}]) and nanosponge (c_{ns} [J kg^{-1} K^{-1}]):

$$Q_{\mathrm{sh}} = (T_{\mathrm{H}} - T_{\mathrm{L}})(c_{\mathrm{re}} w_{\mathrm{re-ads}} + c_{\mathrm{ns}} w_{\mathrm{ns}}) \quad (12)$$

where w_{ns} [kg] is the mass of the nanosponge, and $w_{re\text{-}ads}$ [kg] is the amount of refrigerant adsorbed in nanosponge at T_H. Similarly, a sensible heat Q'_{sh} is supplied from the outside to the condenser, whereas Q'_{sh} is not involved in the calculation of COP. Since temperatures of the two chambers are changed, adsorption and desorption occur at the evaporator and the condenser, respectively (Supplementary Fig. 6b to c). For simplifying, it is assumed that adsorption/desorption occur after the temperatures of the condenser and the evaporator become T_H and T_L, respectively. Adsorption and desorption are associated by the generation of heats, Q_c and Q'_c, respectively, as shown in Supplementary Fig. 6c. Q_c increases the temperature of the target room, but the following process offsets Q_c. By adsorption/desorption, the pressures of the evaporator and condenser become P_2 and P_3, respectively. The order of the pressures is $P_2 < P_1 < P_3$. Next, as shown in Supplementary Fig. 6d, the nanosponge in the evaporator is compressed to desorb the refrigerant, to increase the pressure up to P_1. By this desorption process, the heat (Q_c) is moved from the target room to the evaporator, and the temperature increase at Supplementary Fig. 6c is compensated. The necessary work for this process is W_1 [J], which a part of W_1 turns into heat (Q_{f1}) to warm up the target room

by the internal friction of the nanosponge if it is not perfectly elastic. In the condenser, the nanosponge is freely expanded, and adsorption occurs. The generated heat (Q'_c) is discharged to the outside. When the pressure becomes P_1, the valve is opened, and the nanosponge in the evaporator is further compressed to the limit (Supplementary Fig. 6e). The heat of desorption (Q_L [J]) at this step is used to cool the target room. The necessary work during this step is W_2 [J], while the corresponding heat loss by the internal friction is Q_{f2}. The desorbed vapour at the evaporator migrates to the condenser, and is adsorbed in the nanosponge which is freely expanded. Thus, the heat of adsorption (Q_H) is generated and is discharged to the outside. The desorption amount at the evaporator after the valve is open is denoted as w_{re} [kg]. This is the amount of refrigerant which is used to indeed cool the target room, and is one of the most important parameter to determine COP. Supplementary Fig. 6e is actually the same as the initial state (Supplementary Fig. 6a). By repeating the cycle shown in Supplementary Fig. 6, continuous refrigeration is achieved.

Q_L can be obtained as follows:

$$Q_{\mathrm{L}} = w_{\mathrm{re}} \Delta_{vap} H \tag{13}$$

where $\Delta_{vap}H$ is the enthalpy change of the refrigerant from liquid to gas [J kg^{-1}]. Note that the enthalpy change upon adsorption/desorption is almost the same as that of bulk phase transition.

The total work for the cycle is the sum of W_1 and W_2, and it is denoted as W_{ns}, while the total heat loss by the internal friction (Q_f) is obtained by $Q_f = Q_{f1} + Q_{f2}$. By using equations (12) and (13), COP for this cycle can be described as follows;

$$\begin{aligned}\mathrm{COP} &= \frac{|Q_{\mathrm{L}}| - |Q_{\mathrm{sh}}| - |Q_{\mathrm{f}}|}{|W_{\mathrm{ns}}|} \\ &= \frac{w_{\mathrm{re}}\Delta_{vap}H - (T_{\mathrm{H}} - T_{\mathrm{L}})(c_{\mathrm{re}}w_{\mathrm{re-ads}} + c_{\mathrm{ns}}w_{\mathrm{ns}}) - |Q_{\mathrm{f}}|}{|W_{\mathrm{ns}}|}\end{aligned} \tag{14}$$

In equation (14), W_{ns} depends on the elasticity of nanosponge. The definition of Young's modulus E [Pa] is as follows:

$$E = \frac{\sigma_{\mathrm{f}}}{\varepsilon_{\mathrm{d}}} = \frac{F}{S}\frac{1}{x/L} \tag{15}$$

where σ_f [Pa] and ε_d are stress and strain, respectively. The definition of σ_f is

$$\sigma_{\mathrm{f}} = \frac{F}{S} \tag{16}$$

where F and S are force and cross-section area of nanosponge, respectively. The definition of ε_d is as follows:

$$\varepsilon_{\mathrm{d}} = \frac{x}{L_0} \tag{17}$$

where x is displacement and L_0 is the initial length of nanosponge.

W_{ns} is the sum of the work which is necessary to deform blank nanosponge (W_b) and the work against the force derived from adsorption-induced pressure (W_{ai}).

$$W_{ns} = W_b + W_{ai} \quad (18)$$

By assuming that the Young's modulus is unchanged by the inclusion of refrigerant (see Supplementary Fig. 4), W_b is expressed as follows:

$$W_b = \frac{1}{2}Fx = \frac{1}{2}\frac{S}{L_0}Ex^2 = \frac{1}{2}SL_0E{\varepsilon_d}^2 = \frac{1}{2}V_0E{\varepsilon_d}^2 \quad (19)$$

where V_0 is the initial volume of nanosponge [m^3].

W_{ai} is described as follows:

$$W_{ai} = V_0\varepsilon_d f_{ai} \quad (20)$$

where f_{ai} is the adsorption-induced pressure which can be experimentally determined as shown in Fig 4e. When a nanoporous material expands by adsorption, f_{ai} is described as follows[12]:

$$f_{ai} = f_s - f_0 = -\frac{2\gamma_{sl}}{d_p} + \frac{R_gT\rho_{re}}{M_{re}}\ln\left(\frac{P}{P_0}\right) + (P_0 - P) - f_0 \quad (21)$$

where f_s[Pa], f_0 [Pa] γ_{sl} [N m^{-1}], d_p [m] ρ_{re} [kg m^{-3}], R_g, M_{re} [kg mol^{-1}] are solvation pressure, a prestress at the initial sample volume V_0, the pore wall-liquid surface tension, pore diameter of nanosponge, the density of the adsorbed refrigerant, gas constant, molecular weight of adsorbed refrigerant, respectively. The directions of f_s and f_0 are opposite, and f_0 has a minus value. From equations (20) and (21), W_{aid} is described as follows:

$$W_{ai} = V_0\varepsilon_d\left(-\frac{2\gamma_{sl}}{d_p} + \frac{R_gT\rho_{re}}{M_{re}}\ln\left(\frac{P}{P_0}\right) + (P_0 - P) - f_0\right) \quad (22)$$

When a part of W_b (the ratio is φ_f) is lost as Q_f, $Q_f = \varphi_f W_b$ ($0 \le \varphi_f \le 1$). As shown in Supplementary Fig. 7b, φ_f can be obtained from the hysteresis in stress-strain curves of a nanosponge material.

Accordingly, equation (14) can be deformed as follows:

$$\begin{aligned} \mathrm{COP} &= \left(\frac{\Delta_{vap}Hw_{re} - (T_H - T_L)(c_{re}w_{re-ads} + c_{ns}w_{ns})}{W_{ns}}\right) - \frac{W_b}{W_{ns}}\varphi_f \\ &= 2\left(\frac{\Delta_{vap}Hw_{re} - (T_H - T_L)(c_{re}w_{re-ads} + c_{ns}w_{ns})}{V_0E{\varepsilon_d}^2 + 2W_{ai}}\right) - \frac{W_b}{W_{ns}}\varphi_f \end{aligned} \quad (23)$$

The impact of nanosponge bulk modulus on COP

In equation (23), $w_{re\text{-}ads}$, V_0, w_{re} can be expressed by w_{ns}, using physical properties of nanosponge.

At the state shown in Supplementary Fig. 6a, $w_{\text{re-ads}}$ can be expressed by using w_{ns} as follows:

$$w_{\text{re-ads}} = \rho_{\text{re}}\varphi_{\text{ns}}V_{\text{ns}}w_{\text{ns}} \tag{24}$$

where V_{ns} [m^3 kg^{-1}] is the pore volume of nanosponge and φ_{ns} is the ratio of the pore volume filled with refrigerant. V_{ns} is a physical property of nanosponge. Note that $\rho_{\text{re}}\varphi_{\text{ns}}V_{\text{ns}}$ can be directly determined by the adsorption isotherm measurement at T_{H} (see Supplementary Fig. 7).

V_0 is given by the apparent density of nanosponge ($\rho_{\text{ns-ap}}$ [kg m^{-3}]) and w_{ns} as follows:

$$V_0 = \frac{w_{\text{ns}}}{\rho_{\text{ns-ap}}} \tag{25}$$

The refrigerant-adsorption amounts in the evaporator at Supplementary Fig. 6d and e are denoted as w_{d} and w_{e}, respectively. w_{re} is the difference of them.

$$w_{\text{re}} = w_{\text{d}} - w_{\text{e}} \tag{26}$$

The refrigerant-adsorption amount in the condenser at Supplementary Fig. 6a is $w_{\text{re-ads}}$. From Supplementary Fig. 6b to c, some amount of refrigerant ($w_{\text{b-c}}$) is adsorbed into the nanosponge of the evaporator, generating the heat of adsorption, Q_{c}. At the following compression (from Supplementary Fig. 6c to d), some amount of refrigerant ($w_{\text{c-d}}$) is desorbed and the heat of desorption, Q_{c}, is generated. Thus, the adsorption ($w_{\text{b-c}}$) and the desorption ($w_{\text{c-d}}$) amounts should be the same. Accordingly, w_{d} is equal to $w_{\text{re-ads}}$. Therefore, equation (26) becomes as follows.

$$w_{\text{re}} = w_{\text{re-ads}} - w_{\text{e}} \tag{27}$$

$w_{\text{re-ads}}$ and w_{e} can be obtained from the adsorption isotherm data with and without compression at T_{L} and T_{H}, respectively, as shown in Supplementary Fig. 7a, and thus, COP can be calculated. Alternatively, w_{re} can be roughly estimated by assuming that w_{e} is unchanged at T_{H} and T_{L}. Under this assumption, w_{re} is obtained simply based on the pore-volume difference at Supplementary Fig. 6a and e. With this assumption, the relation between COP and E is derived as follows.

When nanosponge in the evaporator is compressed by ε_{d}, the pore volume of nanosponge becomes from V_{ns} to V_{com} [m^3 kg^{-1}]. On the other hand, the volume occupied by the pore wall ($1/\rho_{\text{ns}}$) is unchanged. Note that ρ_{ns} [kg m^{-3}] is the true density of nanosponge, *i.e.* the density of only the solid component. Thus, V_{com} becomes:

$$V_{\text{com}} = (1 - \varepsilon_{\text{d}})V_{\text{ns}} - \frac{\varepsilon_{\text{d}}}{\rho_{\text{ns}}} \tag{28}$$

w_{re} can be approximated as follows:

$$w_{\text{re}} = \frac{V_{\text{ns}} - V_{\text{com}}}{V_{\text{ns}}} w_{\text{re-ads}} = \rho_{\text{re}}\varphi_{\text{ns}}w_{\text{ns}}\left(V_{\text{ns}} + \frac{1}{\rho_{\text{ns}}}\right)\varepsilon_{\text{d}} \tag{29}$$

Actually, w_{e} is larger at T_{L}, and therefore, w_{re} is overestimated under the above approximation.

There is the following relation between the apparent density of nanosponge ($\rho_{\text{ns-ap}}$ [kg m^{-3}]), ρ_{ns}, and

V_{ns}:

$$\rho_{ns-ap} = \left(\frac{1}{\rho_{ns}} + V_{ns}\right)^{-1} \tag{30}$$

Therefore, equation (26) becomes

$$w_{re} = \frac{\rho_{re}\varphi_{ns}}{\rho_{ns-ap}} w_{ns}\varepsilon_d \tag{31}$$

From equations (23), (24), (25), and (31), it is found that COP of RMPTA does not depend on the amount of nanosponge (w_{ns}).

Hereafter, the approximate relation between COP, E, and ε_d is obtained when water is used as a refrigerant. In equation (23), $\Delta_{vap}H$ and c_{re} are 2453 kJ kg^{-1} and 4.184 kJ kg^{-1} K^{-1}, respectively. $T_H - T_L$ is assumed to be 20 K. For c_{ns}, the value of graphite (0.72 kJ kg^{-1} K^{-1}) can be assumed. φ_f is measured to be 0.25 in a ZTC/PTFE sheet, and this value is used for the rough calculation here. Thus, equation (23) becomes

$$\mathrm{COP} = 2\left(\frac{2453000w_{re} - 20(4184w_{re-ads} + 720w_{ns})}{V_0E\varepsilon_d{}^2 + 2W_{ai}}\right) - 0.25 \tag{32}$$

To obtain W_{ai} given by equation (21), the following parameters are used: $\gamma_{sl=}$ 94 mN m^{-1} and $T = 298$ K. f_0 can be determined to be −0.18 GPa, when the deformation of ZTC is 0 as shown in supplementary Fig. 9. Therefore,

$$W_{aid} = E_{aid}V_0\varepsilon_d,$$
$$E_{aid} = -\frac{0.19}{d_p} + 1.38 \times 10^8 \ln\left(\frac{P}{P_0}\right) + (P_0 - P) + 1.8 \times 10^8 \tag{33}$$

By introducing equations (24), (25), (31) and (33) into (32),

$$\mathrm{COP} = \frac{2\rho_{ns-ap}}{E\varepsilon_d{}^2 + 2E_{aid}\varepsilon_d}\left(2453000\frac{\rho_{re}\varphi_{ns}}{\rho_{ns-ap}}\varepsilon_d - 83680\rho_{re}\varphi_{ns}V_{ns} - 14400\right) - 0.25 \tag{34}$$

In equation (34), ρ_{re} is 1000 kg m^{-3}. $\rho_{ns\text{-}ap}$ of general porous materials is at most ca. 1000 kg m^{-3}. V_{ns} can be assumed as at most about 0.003 m^3 kg^{-1}. P is assumed as 2.9 kPa (the same as that in Supplementary Fig. 7 and 8). φ_{ns} is assumed to be 1. Accordingly, COP can be approximately expressed by E and ε_d, as shown below:

$$\mathrm{COP} = \frac{1}{E\varepsilon_d - 2\left(\frac{0.19}{d_p} - 1.68 \times 10^8\right)}\left(4.91 \times 10^9 - \frac{5.31 \times 10^8}{\varepsilon_d}\right) - 0.25 \tag{35}$$

Equation (35) is used for the calculation of Fig. 6g.

References

1. Ito, M. *et al.* Reversible pore size control of elastic microporous material by mechanical force. *Chem.-Eur. J.* **19**, 13009-13016 (2013).
2. Endo, A. *et al.* Water adsorption–desorption isotherms of two-dimensional hexagonal mesoporous silica around freezing point. *J. Colloid Interface Sci.* **367**, 409-414 (2012).
3. CP2K Open source molecular dynamics. http://www.cp2k.org.
4. William, L. J. *et al.* Comparison of simple potential functions for simulating liquid water. *J. Chem. Phys.* **79**, 926-935 (1983).
5. Rappe, A. K. *et al.* UFF, a full periodic table force field for molecular mechanics and molecular dynamics simulations. *J. Am. Chem. Soc.* **114**, 10024-10035 (1992).
6. Peterson, B. K. & Gubbins, K. E. Phase transitions in a cylindrical pore. *Mol. Phys.* **62**, 215-226 (1987).
7. Plimpton, S. Fast parallel algorithms for short-range molecular-dynamics. *J. Comput. Phys.* **117**, 1-19 (1995).
8. Stuart, S. J., Tutein, A. B. & Harrison, J. A. A reactive potential for hydrocarbons with intermolecular interactions. *J. Chem. Phys.* **112**, 6472-6486 (2000).
9. Nishihara, H. *et al.* Graphene-based ordered framework with a diverse range of carbon polygons formed in zeolite nanochannels. *Carbon* **129**, 854-862 (2018).
10. Moran, M. J. & Shapiro, H. N., *Fundamentals of engineering thermodynamics*. (John Wiley & Sons, 2006).
11. Wylen, G. J. V. & Sonntag, R. E., *Fundamentals of classical themodynamics 3rd ed*. (John Wiley & Sons, 1986).
12. Gor, G. Y. & Neimark, A. V. Adsorption-induced deformation of mesoporous solids. *Langmuir* **26**, 13021-13027 (2010).
