# Supplementary material for: Force-driven reversible liquid–gas phase transition mediated by elastic nanosponges
Source: Nat Commun. 2019 Jun 17;10:2559. doi: 10.1038/s41467-019-10511-7 (PMC6572794; doi:10.1038/s41467-019-10511-7)
Supplement: Supplementary file 3 — Description of Additional Supplementary Files [file 41467_2019_10511_MOESM3_ESM.docx]

**Description of Additional Supplementary Files**

**File Name: Supplementary Movie 1**

**Description:** In situ SEM movie during pressing of a single grain of ZTC.

**File Name: Supplementary Movie 2**

**Description:** In situ SEM movie during pressing of a single grain of AC.

**File Name: Supplementary Movie 3**

**Description:** MD simulation of forced desorption of H2O adsorbed by ZTC at 298 K.

**File Name: Supplementary Movie 4**

**Description:** MD simulation of forced desorption of H2O adsorbed by ZTC at 350 K.
